# The Aged Intestine: Performance and Rejuvenation

**DOI:** 10.14336/AD.2021.0202

**Published:** 2021-10-01

**Authors:** Qiwen Wang, Yadong Qi, Weiyi Shen, Jilei Xu, Lan Wang, Shujie Chen, Tongyao Hou, Jianmin Si

**Affiliations:** ^1^Department of Gastroenterology, Sir Run Run Shaw Hospital, School of Medicine, Zhejiang University, Hangzhou 310016, Zhejiang Province, China.; ^2^Institute of Gastroenterology, Zhejiang University, Hangzhou 310016, Zhejiang Province, China.

**Keywords:** aging, intestine, intestinal microbiota, bile acids, short-chain fatty acids

## Abstract

Owing to the growing elderly population, age-related problems are gaining increasing attention from the scientific community. With senescence, the intestine undergoes a spectrum of changes and infirmities that are likely the causes of overall aging. Therefore, identification of the aged intestine and the search for novel strategies to rescue it, are required. Although progress has been made in research on some components of the aged intestine, such as intestinal stem cells, the comprehensive understanding of intestinal aging is still limited, and this restricts the in-depth search for efficient strategies. In this concise review, we discuss several aspects of intestinal aging. More emphasis is placed on the appraisal of current and potential strategies to alleviate intestinal aging, as well as future targets to rejuvenate the aged intestine.

Because of the growing elderly population, age-related healthcare issues are gaining increasing interest from the scientific community. Recent advances highlight efficient strategies, such as diet restrictions[1], rapamycin[2], and nicotinamide adenine dinucleotide (NAD) replacements [3] to prevent and ameliorate the overall dysregulation in various aging processes.

The intestine is considered a pervasive and important player involved in an array of biological events, including digestion, absorption, and immune modulation. Its wide-ranging function determines its profound impact on overall health [4,5]. Similar to other body organ systems, the intestine also undergoes senescence. Increasing age enhances intestinal disease incidence, such as malnutrition [6], chronic constipation [7], and colorectal cancer [8]. Central to understanding the underlying mechanisms, is to clarify age-related changes in commensal microbiota, the immune system, intestinal stem cell (ISC), the epithelial function, and the enteric nervous system (ENS). These changes not only account for localized gastroenterology disorders, but are associated with a decline in multiple systems throughout the body, including the nervous [9, 10], cardiovascular [11], endocrine [12-14], and skeletal [15] systems (Fig.1). Therefore, it is important to explore strategies to rescue the aged intestine. Over the last few decades, a vast number of studies have shown the capacity to delay intestinal aging through a variety of mechanisms. However, it is still far from our aspiration of strategies with high and all-round effects against intestinal aging, as well as security.

The advancement of metabolomics, in conjunction with transcriptomics and proteomics, expands our knowledge on the aged intestine. Results from a metabolomic analysis serve as a reservoir from which to identify novel targets against intestinal aging. On the contrary, an unprecedented number of intrinsic and extrinsic compounds pose an enormous challenge for accurate verification.

In this review, we describe changes occurring in the aged intestine, including dysbiosis, immune imbalance, stem cell exhaustion, barrier dysfunction, and enteric neurodegeneration. Additionally, particular focus is placed on strategies and potential molecular targets that contribute to the alleviation of intestinal aging.

**Figure 1.**
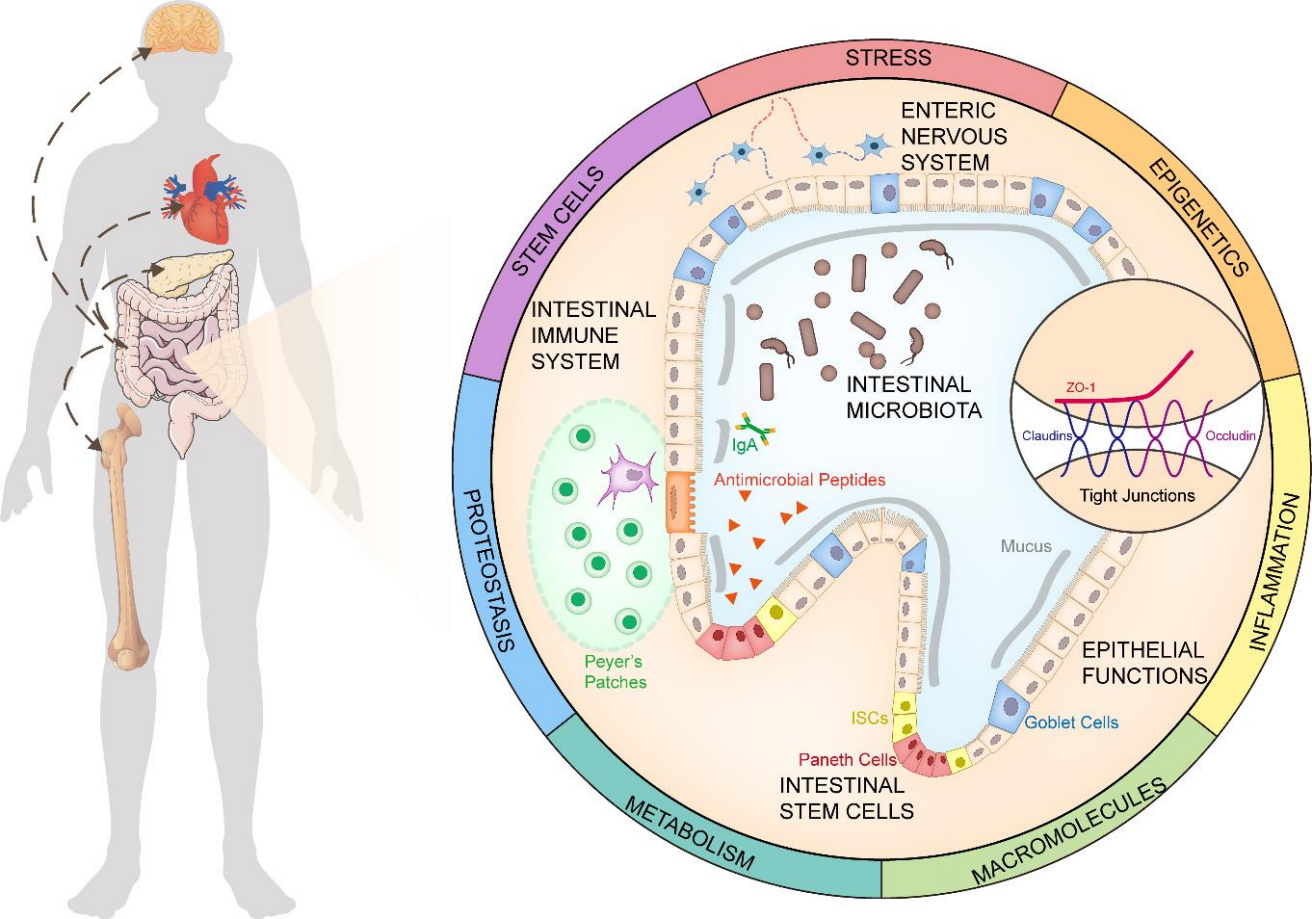
The Age-related Changes in the Intestine. During senescence, the intestine gains changes in terms of the intestinal microbiota, immune system, intestinal stem cells, epithelial functions, and the enteric nervous system. These changes in the aged intestine are responsible for many overall age-related diseases, such as the brain, heart, bone, and endocrine system. The Geroscience perspective that makes it more comprehensive to understand anti-aging mechanisms, would enlighten us on the development of strategies to rejuvenate the aged intestine.

## 1. Age-related Changes in the Intestine

Intestinal function has a profound impact on aging. In terms of longevity, for example, the intestine of *Caenorhabditis elegans* is the key longevity signaling center that is signaled by the brain and further propagates longevity signals to other tissues in the body[4]. As for progeroid mice, their lifespan can be prolonged through the transplantation of fecal microbiota in wild-type mice [5]; therefore, indicating that commensal microbiota inside the intestine can serve as a target against aging. Moreover, several studies have confirmed a strong association between the gastrointestinal tract and age-related complications, such as Alzheimer’s disease [9], cognitive decline [10], obesity and insulin resistance [12-14], cardiovascular disorder [11], arthritis [15], and overall frailty[16], in other organs and systems.

Besides disturbing overall health, the aged intestine also suffers from morphological and functional changes over time. These relationships have been well established between aging and the intestinal age-related changes, including alterations in the microbiota, immune system, ISCs, epithelial functions, and ENS. Further studies show interactions among these changes. Thus, fully understanding the complex crosstalk between intestinal age-related changes and aging, is essential to develop intestinal aging interventions.

### 1.1 Morphological and Functional Changes in the Aged Intestine

The intestine is an important digestive apparatus in digestion and absorption across species. Its morphology and function vary with age, which has been supported by studies in several models and humans.

Combining transmission electron microscopy with confocal microscopy with 3-D volumetric reconstructions, McGee et al. illustrated loss of intestinal nuclei and microvilli of aging *C. elegans*, in addition to increased variability of the shape and size of the intestinal lumen that age-related germline swelling was partly blamed for [17]. An abnormal intestinal structure also occurs in aging mammals. Morphological changes in aged mouse and rat models have been described as thicker muscular layers, distorted villi, more secretory Paneth and goblet cells, and impaired junctions between adjacent enterocytes [18-24]. Mice have wider and higher villi with increasing age [22], while rats have wider and shorter ones [23]. Furthermore, rats have darkly stained nuclei in their aged intestines [23]. Human studies showed no significant changes in the duodenum [25]. However, abnormal hyperproliferation and apoptosis were found in enterocytes of a normal elderly group, which resulted in impaired function of the aged intestine [26, 27].

In rodents, the intestinal functions are also affected by aging, including degenerative digestion and absorption [28], as a result of the reduced activity [29] or lower production [30] of related enzymes. In aged mice, impaired adaptive mechanisms to diet were also found [31]. However, researchers have rarely reported changes in intestinal secretions and absorption in elderly people [32]. Nevertheless, multiple studies have confirmed humans share similar age-related changes with rodents, ranging from reduced absorption [33] to lower digestive secretions[34], and declined motility [35]. Although more evidence is needed to clarify the conflicting results, the idea is still acceptable that the elderly suffer from an elevated incidence of gastrointestinal disorders such as associated cancer [8] and infections [36] (comprehensively reviewed by Dumic *et al*.[37]).

### 1.2 The Intestinal Microbiota

Bacteria, fungi, protozoa, and viruses are located in the intestine at high quantities. They play critical roles in human physiology and disease, due to their abilities to limit pathogenic growth, ferment food, as well as produce mucus and lipid metabolites[38]. As reported, gut microbes vary with age, not only in terms of composition imbalances [39], such as fewer *Bacteroidetes* and more *Firmicutes* [40], but also in terms of degraded intrinsic functions, such as evolution and mutations [41]. A study containing four age groups, covering almost the entire adult lifespan, showed the sustained reduction of particular microbiota with age [42]. Intriguingly, among the healthy aging people, aged 70-82 years, and an elderly cohort with diabetes or other age-associated disorders, no gut microbiome changes were observed, except the proportion of the genus *Akkermansia* [43]. Together, both indicated that the altered microbiome may account for senescence itself rather than age-related infirmities, with bacterial taxa contributing to respective disorders.

Besides expanding lifespan [5], the imbalances of intestinal microbiota can induce or reduce aging[39], and age-related illnesses [44,45]. Current studies confirmed that only in combination with dysbiosis, can the diminished intestinal barrier lead to systemic inflammation [46] This is a key hallmark and driver of senescence [47], supporting the indispensable importance of gut flora in aging. An intestinal microbiota that was reported to change with age, *Akkermansia muciniphila*, has been linked to colitis-associated tumorigenesis [48] and cancer therapy [49], indicating the relationship between age-associated changes in intestinal microbiota and the elevated incidence of cancer.

However, the general mechanisms by which microbiota affects the host are still unclear. It is widely accepted that the consequent changes in microbiota-derived metabolites, especially small-molecule metabolites, could be the main contribution of microbiota to host biology [50], which lays a preliminary theoretical foundation for the investigation of bacterially derived metabolites against intestinal aging.

### 1.3 The Intestinal Immune System

“Immunosenescence” refers to immune changes related to poor clinical outcomes in the elderly compared to that in young individuals, such as inflammageing [51,52]. Interleukin (IL)-10-producing T follicular helper cells [53] and the imbalance of immunological mediators [52] are involved in this systemic immune degeneration.

The intestinal immune system is the largest immune compartment, consisting of gut-associated lymphoid tissues (GALT) as well as effector cells. Its mucosal immune responses are important for defense against pathogens, including antigen uptake by M cells, presentation in Peyer’s patches, differentiation and migration of B immunoblasts, and production and transport of antibodies [54]. Over the years, the intestine experiences immune degeneration and aggravates systemic aging. Aging impairs the migration of IgA immunoblasts [54] to the intestinal lamina propria and lowers antibody titers [55], resulting in a diminished mucosal immune response [56]. Consequently, it is more common for the elderly to suffer from bacterial or viral gastrointestinal infections [36]. In addition, aging reshapes the gut microbiota, making it a modulator of age-related changes in the immune system. For example, the age-related decline in *Firmicutes* and an increase in *Enterobacteriaceae* exacerbate inflammageing [57]. The additional consumption of tryptophan by aged gut microbiota is speculated to enhance inflammation in centenarians [58].

### 1.4 Intestinal Stem Cells

For maintaining tissue homeostasis, ISCs are highly active in supporting the repair of damaged tissues, and the continuous and rapid cell turnover of intestine [59, 60]. As long as rapid replication occurs, ISCs are exposed to age-related risks. The features of aged ISCs are identified as altered numbers and declined functions. No conflicts have been reported in the weakening regenerative capacity [24, 61], but the quantitative issue is unclear. In old *Drosophila*, the intestinal epithelium exhibits an increased number of proliferating cells [62]. However, there are competing data in aged mice. No change in the established marker Lgr5 ISCs has been reported [24], while others had an increased population of cells expressing sub-low SOX9 [61], a marker of progenitor cells in old mice [63]. At the mercy of biomarkers selected for investigation, different studies exhibit competing changes in the quantity variance of ISCs upon aging. Some researchers have described it as a constant absolute number of ISCs with alternative expression of markers [24]. However, the overall understanding of the effect of aging on ISCs is awaiting more data, especially human studies. On the contrary, the loss of regulation of ISC proliferation for self-renewal results in disrupted organ homeostasis and impaired self-repair function after damage, even shortening lifespan[64].

**Figure 2.**
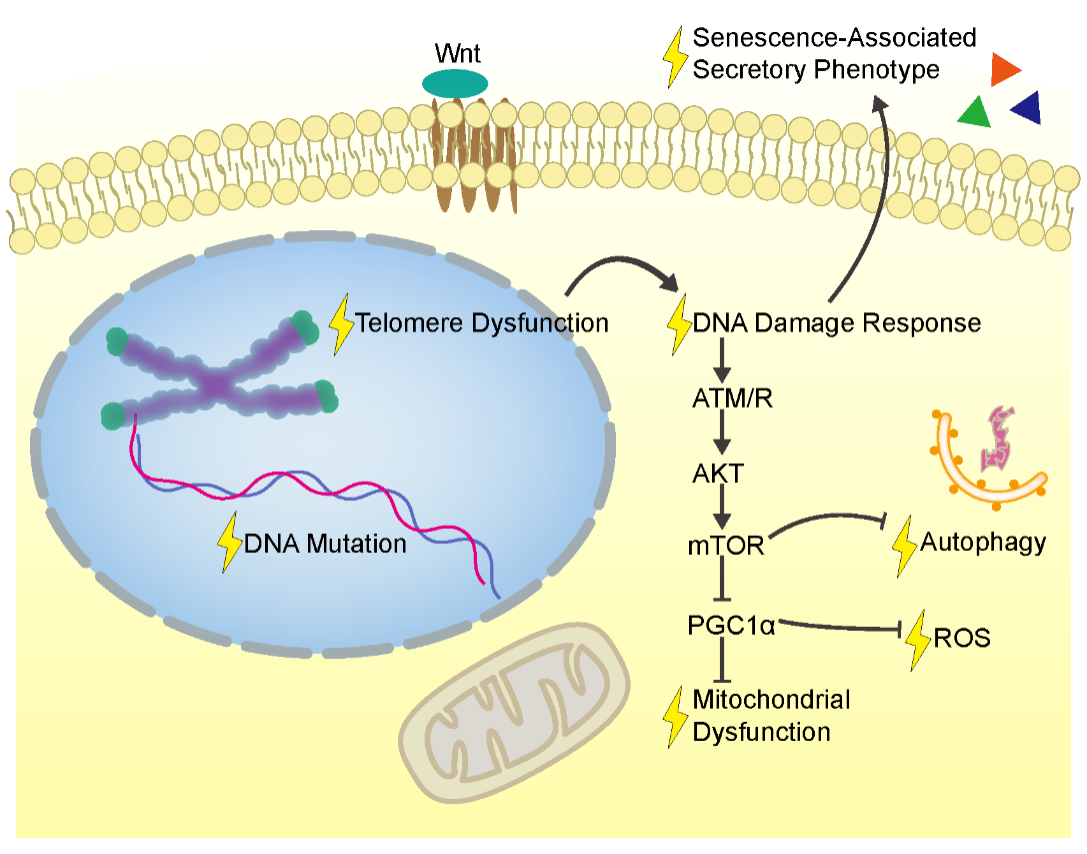
The Factor Network Contributing to the Intestinal Stem Cell Aging. An intracellular and extracellular network contributes to the senescence of ISCs, including telomere dysfunction, DNA damage response, DNA mutation, mitochondrial dysfunction, oxidation stress, autophagy dysregulation, and senescence-associated secretory phenotype. Several cellular signaling pathways are also involved, such as the Wnt and mTOR pathways. ATM/R, protein kinases ATR and ATM; AKT, protein kinase B; mTOR, mammalian target of rapamycin; ROS, reactive oxygen species; PGC1α, peroxisome proliferator activated receptor-γ co-activator 1-α.

Both cell-intrinsic and extrinsic factors contribute to ISC aging. Telomere dysfunction induced by ISC replication [65] triggers DNA damage response (DDR) pathways [66] and mitochondrial dysfunction [67]. DDR further activates the innate immune response as a senescence-associated secretory phenotype (SASP) [68], which spreads senescence to neighboring ISCs [65, 66] in a paracrine manner [69]. Mitochondrial dysfunction, including impaired lipid metabolism [70, 71] and mitophagy [72] produces oxidative stress to accelerate aging. Accumulation of DNA mutations by age drives the dysregulation of ISC proliferation [73]. Abnormalities in cell signaling pathways, such as target of rapamycin (TOR) and Wnt also participate in aged ISCs [74] (Fig.2).

Intestinal cancer is strongly associated with age and originates mainly from ISCs [75]. Although more work is required to elucidate the importance of age-related risks in the carcinomatous transformation of ISCs, rejuvenation of ISCs, including remodeling related signaling pathways such as Wnt signaling, could be a promising strategy to revive the aging intestine, at least reducing age-related intestinal cancer.

### 1.5 Intestinal Epithelial Barrier Function

The integrity of the intestinal epithelial barrier function requires a contiguous cell layer, an intracellular junctional complex of molecules [76], expression of mucus, defensin secretion from multiple cells with respective functions [77], including ISCs, immune cells, and goblet cells, which is pivotal to ensure that the intestine is a semipermeable membrane that exerts the admission of nutrients and the prevention of pathogens or toxins. Age-related intestinal barrier dysfunction in elderly organisms can be observed in species varying from rats [78] to baboons [79]. In two cohorts of healthy adults, the older group exhibited increasing levels of zonulin, an intestinal permeability biomarker [80].

The age-related increase in gut permeability accounts for chronic and systemic mild inflammatory responses [81] that accelerate aging in mammals [82]. This inflammation, as above, arises owing to dysbiosis [46]. In the context of dysbiosis, the degraded intestinal barrier permits the translocation of gut contents, such as bacteria and their products, into the circulatory system [46], which shortens the lifespan of *C. elegans* [76], and foreshadows the death of *Drosophila* [83].

### 1.6 Enteric Nervous System

Considered as the second brain, the ENS consists of more than 500 million neurons to form the myenteric and submucosal plexus [84]. Similar to the brain, ENS suffers from neurodegeneration during aging, which is a cause of constipation in the elderly [85]. The prevalence of constipation in patients with Parkinson’s disease [86] supports this idea. The accumulation of age witnesses a significant loss of enteric neurons [87], especially choline acetyltransferase positive ones [88]. Whether a decrease in the neuron density or the decreasing number played a more prior role [89], we could highlight the age-related changes of ENS. However, except for 5-hydroxytryptamine (5-HT) [90], more therapeutic approaches to protect ENS from aging are vague.

Collectively, age-related phenomena and mechanisms of the intestine rely on the combination of gut microbiota, immune system, ISCs, intestinal barriers, and ENS, but more efforts are needed to better understand these mechanisms. From the Geroscience perspective, aging research was conducted in seven areas: adaptation to stress, epigenetics, inflammation, macromolecular damage, metabolism, proteostasis, and stem cell exhaustion [91]. A comprehensive view about age-related intestinal changes also covers oxidative stress responses [92], genomic modifications [73], heterochromatin maintenance [93], lipid metabolism [70], mitophagy and autophagy[94], *etc* (Fig.1).

It should be noted that the degeneration phenomena differ among organisms. Taking intestinal architecture as an example, a quantitative histology performed on patient jejunal biopsy specimens showed no significant differences in surface to volume ratios and enterocyte height between elderly patients and the younger ones [95]. In the mouse intestine, aging causes a decreased number of crypts; however, an increase in the number of cells per crypt, in addition to an elevated villus height [24]. The same issue also arised, when the age-related trend of the ISC number across species was identified [24, 61, 62]. More data are required to delineate the complex changes in the human intestine during aging to select targeted aging models.

## 2. Strategies to Rejuvenate the Aged Intestine

The intestine is a key interface between the host and nutrient substances or microbiota. Several studies are emerging showing that appropriate means, such as diet control and pharmaceutical intervention, motivate people to fight against intestinal aging. Furthermore, the changes in intestinal and microbial metabolites caused by these means could be valuable to the interpretation of the underlying mechanisms. In this section, we discuss diet regimens and pharmaceutical interventions as well as metabolites derived by the host and microbiota to elucidate on the potential strategies to rescue the aged intestine.

### 2.1 Dietary Restriction Regimens

Dietary restriction regimens (DR) such as caloric restriction, ketogenic diet, and intermittent fasting, are strongly proven anti-aging interventions in a wide range of species [96]. Multiple mechanistic pathways are involved in its effects on expanding lifespan and alleviating age-related diseases, such as hindering oxidative damage, suppressing TOR, and the insulin/insulin-like growth factor 1 (IGF-1) pathway [97]. In the intestine, however, DR impairs the mucus in the small bowel and decreases the number of several cells in gut-associated lymphoid tissue, which was intensely reviewed by Genton [98] indicating the possible harmful effects of DR on the intestine. On the contrary, by rebalancing apoptosis with intestinal cell repair, DR enhances the intestinal barrier in *Drosophila* [99]. In mice, DR boosts ISC competition to drive-out fewer fit cells, with the mutation retention decreasing [100]. The effect of DR on ISCs brings hope for cancer prevention and aging postponement in the intestine. In addition, DR provides mice with alternative microbiota as well as altered fecal metabolites [101]. In short, DR plays differential roles in various aspects of intestinal aging and requires further assessment.

### 2.2 Resveratrol

Resveratrol (RSV), a natural non-flavonoid polyphenolic compound, is widely found in food such as wine and mulberries [102]. Since the report about RSV extending the lifespan of *Saccharomyces cerevisiae* as a remarkable stimulus of Sirtuin1 (SIRT1) [103], numerous studies on the anti-aging benefits of RSV have emerged over the last decade. Through adenosine 5’-monophosphate-activated protein kinase (AMPK), Sirtuins, and AKT, RSV contributes to anti-oxidant, anti-inflammation [104], anti-infection [105], calorie restriction mimetic, telomere maintenance [104], mitochondrial fission [106], and endoplasmic reticulum stress (ER stress) [107], thus preventing aging in multiple body systems [104]. At the same time, AMPK [108] and SIRT1 [109] signaling pathways are the underlying mechanisms of RSV mitigating adult stem cell aging, in addition to activating nuclear factor erythroid-2-related factor 2 (Nrf2) [110]. The scientific community pays more attention to the anti-inflammatory effects in the intestine. Dozens of drug-induced colitis studies in animals have pushed forward research on RSV and human inflammatory bowel disease [102]. RSV also confers intestinal permeability benefits by increasing tight junction protein expression. In mice fed with a high fatty diet, resveratrol co-administration was found to improve dysbiosis and the leaky gut by impairing the loss of tight junction protein, and then ameliorate systemic inflammation and endotoxemia [111]. Furthermore, the current study in the highly fatty-diet rats showed that it was the gut endocannabinoid system that mediated the maintenance of intestinal barrier function by RSV[112]. Furthermore, knockdown of Nrf2, as well as inhibition of PI3K/AKT, abolished the RSV-induced increase of tight junction protein expression against oxidative stress [113], indicating that more mechanisms remain to be explored. By demonstrating that RSV rehabilitates the debris of villus structures and goblet cells by heat-stress responses [114], a study on black-boned chickens highlighted the potential benefits of RSV on the morphological changes in the aging intestine.

### 2.3 Metformin

**Figure 3.**
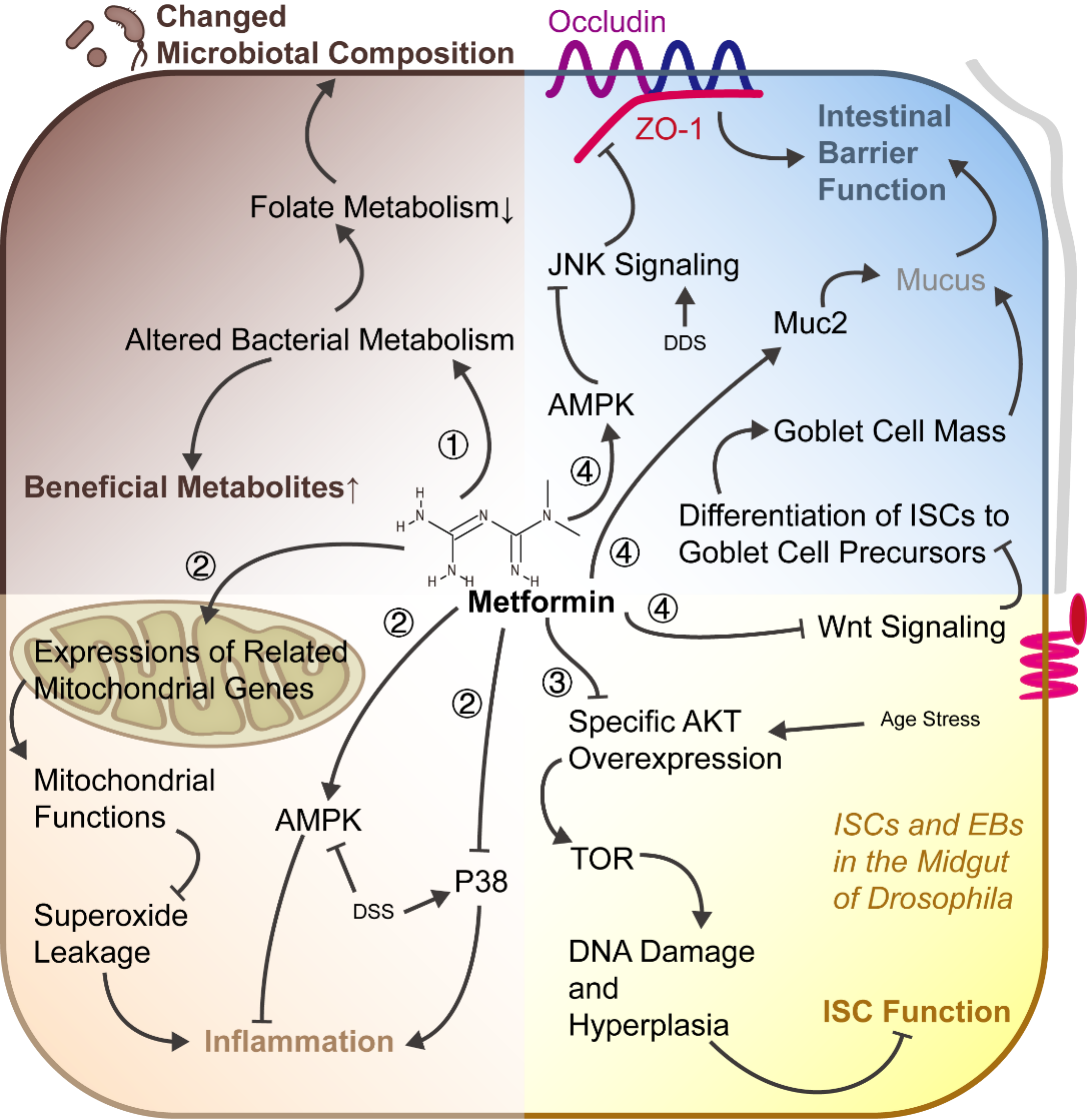
An integral view on the anti-aging effect of metformin in the intestine. Four main changes take place in the aging intestine, including the intestinal microbiota, immune system, ISCs, and epithelial functions. Metformin exerts its integral effects to mitigate age-related changes in the intestine. ?Metformin alters bacterial metabolism to improve the production of beneficial metabolites, as well as to interfere with folate metabolism, which leads to a changed microbial composition. ?The anti-inflammatory effect of metformin is mediated by the regulation of mitochondrial gene expression, activation of AMPK, and inhibition of P38. ?Through attenuating the age-related specific AKT overexpression, metformin relieves DNA damage and ISC hyperplasia in the midgut of *Drosophila*, favoring homeostasis of ISCs. ?Tight junctions and mucus produced by goblet cells are both important components in the maintenance of the intestinal barrier. The inhibition of JNK signaling and Wnt signaling by metformin contributes to the expression of tight junction proteins and the differentiation of ISCs to goblet cells, which, accompanied by a metformin-induced increase in Muc2 expression, reinforces the intestinal barrier. AMPK, adenosine 5’-monophosphate-activated protein kinase; DSS, dextran sulfate sodium; AKT (PKB), protein kinase B; TOR, target of rapamycin; ISC, intestinal stem cell; EB, enteroblast; JNK, c-Jun N-terminal kinase.

Metformin, the prescribed oral antidiabetic therapy, delays aging in *C. elegans* [115] and mice [116], with beneficial effects on diabetes, cognitive function, and cancer in humans, and is involved in the complex of IGF-1, mTOR, AMPK, regulation of reactive oxygen species (ROS) production, and DNA damage [117], and age-related cellular processes such as mitochondrial function, ER stress [107], inflammation, autophagy, and cellular senescence [117]. As for intestinal aging, metformin is considered sufficient to mitigate restoration-related deterioration in a variety of ways (Fig.3). Metformin remodels the metabolism of intestinal bacteria to retard aging, that is accounted for by altered microbial folate metabolism [115] and the increased yield of beneficial microbial productions by metformin [118]. Administration of metformin activates AMPK and inhibits P53, leading to less colonic pathological inflammation [119]. Moreover, improvement of superoxide leakage by increasing the expression of related mitochondrial genes is another efficient way for metformin to inhibit chronic inflammation [116]. A series of studies in the *Drosophila* midgut, revealed that metformin inhibits ISC aging, described as hyperproliferation, by improving DNA damage and genomic instability [120], further being accounted for by AKT/TOR signaling modulation [121] and Atg6-dependent autophagy [122]. In the mouse intestine, metformin treatment recovers the tight junction protein expression abated by a high liquid controlled diet [123], as well as lipopolysaccharide (LPS) [124], where in part an AMPK/JNK-dependent signaling pathway participates [125]. By modulating the differentiation of ISCs in older mice by suppressing Wnt signaling, metformin raises the number of goblet cells, in which metformin further increases Muc2 [118]. The combined action on tight junctions, ISCs, and goblet cells endows metformin with the ability to reinforce the intestinal barrier. The restoration of autophagy and NAD levels in senescent cells [126] contributes to a more comprehensive understanding of metformin in aging.

### 2.4 Bile Acids

Bile acids (BAs), small steroid molecules synthesized in the liver and modified by intestinal microbiota, are of various kinds. What distinguishes between BA types in terms of molecular structure lies in the existence, position, and conformation of the hydroxyl group, in addition to the binding of taurine or glycine, that are responsible for diverse extensions of solubility, metabolism processes, and physiological functions [127]. A recent study reported extraordinary BAs conjugated to phenylalanine and leucine, denoting a continually rising number of BA types, as more data emerge [128]. Despite individual differences in the composition of BAs in feces, unconjugated BAs account for the major components, such as deoxycholic acid (DCA) and lithocholic acid (LCA) [129]. Through activation or resistance, especially the farnesoid X receptor (FXR), transmembrane G protein-coupled receptor 5 (TGR5 or GPBAR1), pregnane X receptor (PXR), and vitamin D receptor (VDR), BAs take part in various physiological processes such as synthesis modulation of their own, lipid absorption, metabolism, and the immune system [127].

**Table 1:** Age-related Changes of Bile Acids.

	Total BA	Unconjugated BAs		Conjugated BAs		Ref
Rat Bile	INCREASED	DECREASED: αMCA, βMCA, ωMCA, CA, CDCA, DCA, UDCA		INCREASED: T-CA, T-MCA DECREASED: G-DCA, G-LCA UNCHANGED: G-UDCA, G-CDCA, T-UDCA, T-CDCA, T-DCA, T-LCA		[132]
Male Mouse Serum	UNCHANGED	Concentration: INCREASED: UDCA INCREASED and DECREASED therefore: βMCA, CA, HDCA UNCHANGED: CDCA, DCA	Proportion: INCREASED: βMCA, HDCA, UDCA DECREASED: CDCA, DCA	Concentration: UNCHANGED: T-αMCA, T-βMCA, T-CA, T-HDCA, T-UDCA	Proportion: INCREASED: T-βMCA, T-MDCA DECREASED: T-DCA, T-HDCA	[133]
Female Mouse Serum	INCREASED	Concentration: INCREASED: βMCA, CDCA, DCA UDCA UNCHANGED: CA, HDCA	Proportion: INCREASED: βMCA DECREASED: DCA, HDCA	Concentration: INCREASED: T-βMCA, T-ωMCA, T-UDCA UNCHANGED: T-αMCA, T-CA, T-DCA, T-HDCA, T-MDCA	Proportion: INCREASED: T-βMCA, T-UDCA DECREASED: T-DCA, T-MDCA	
Male Mouse Liver	UNCHANGED	Concentration: INCREASED and DECREASED thereafter: βMCA UNCHANGED: αMCA, CDCA, DCA, HDCA, LCA, UDCA	Proportion: INCREASED: βMCA DECREASED: ωMCA, CA, DCA, HDCA, MDCA, LCA, UDCA	Concentration: INCREASED: T-αMCA, T-βMCA DECREASED: T-DCA UNCHANGED: T-ωMCA, T-CA, T-CDCA, T-HDCA, T-MDCA, T-LCA, T-UDCA	Proportion: INCREASED: T-αMCA, T-βMCA DECREASED: T-ωMCA, T-DCA, T-LCA	
Female Mouse Liver	UNCHANGED	Concentration: INCREASED: CA INCREASED and DECREASED thereafter: ωMCA UNCHANGED: αMCA, CDCA, DCA, HDCA, LCA, UDCA	Proportion: DECREASED: αMCA, DCA, HDCA, LCA, MCA, MDCA	Concentration: INCREASED and DECREASED thereafter: T-αMCA, T-βMCA, T-CDCA, T-LCA, T-MDCA, T-UDCA UNCHANGED: T-ωMCA, T-CA, T-DCA, T-HDCA	Proportion: INCREASED: T-αMCA, T-βMCA DECREASED: T-DCA, T-HDCA, T-LCA, T-MDCA	

BA, bile acid; MCA, Muricholic acid; MDCA, Murideoxycholic acid; CA, cholic acid; CDCA, Chenodeoxycholic acid; DCA, Deoxycholic acid; HDCA, Hyodeoxycholic acid; LCA, lithocholic acid; UDCA, Ursodeoxycholic acid; T-, Taurourso-; G-, Glyco-.

An analysis of metabolites in a Chinese cohort exhibited a higher level of total BAs in the feces of centenarians [130]. Changes take place in the reabsorption of BAs with advanced aging, rather than in biliary secretion [131]. Age-related alterations of the BA profile have been reported in rats [132] and mice [133] (Table 1), albeit in the absence of features of the elderly ones. Moreover, metabolomic analysis showed altered BA profiles accompanied by age-related dysbiosis in *Lmna^G609G/G609G^* and *Zmpste24^-/-^* mice, two typical progeroid animal models [5, 134], among which potentially anti-aging BAs would be discussed as follows.

The altered BA profiles play double-edged roles during aging. On the one hand, BAs exert beneficial effects in attenuating metabolic disorders [135], cardiovascular disease [136, 137], impairment of the nervous system function [138, 139], and deterioration of cartilage [140] and bone [141] common in the elderly. Functions of the BA receptor are also involved in age-related mechanisms or signaling pathways, such as AMPK [141, 143], Nrf2 [144], and autophagy [145]. On the other hand, cytotoxicity [146] and tumor promotion [147, 148] of BAs call our attention to prudent assessment of the situation of BAs in aging. In view of different natures, alongside different age-related trends of BAs, it is imperative to identify the anti-aging effects of a special kind of BA separately.

In animal experiments, cholic acid (CA), whose activation ability to related receptors is weaker than that of others [127], is frequently administered. A diet enriched with CA extends lifespan and alleviates weight loss associated with intestinal aging in progeroid mice [134]. However, the mechanism behind this effect remains to be explored. DCA and LCA show biological toxicity due to their strong hydrophobicity. Such toxicity, on the contrary, means inhibition of both the infection [149] and growth of a tumor [148, 150], which disturbs the elderly. Previous studies have revealed the anti-aging effect of LCA in yeast and worms mediated by a mitochondria-centered mechanism, including remodeling lipid and carbohydrate metabolism, and attenuating mitochondrial network fragmentation [151, 152]. In addition, LCA has a unique effect of activation of VDR to augment tight junction proteins, preventing and ameliorating intestinal epithelial barrier injury [153]. The current study showed that incubation with bile extract or LCA promoted mouse intestinal organoid growth via activation of TGR5 in ISCs. Furthermore, elevating endogenous BAs by intraperitoneal injection of cholecystokinin contributes to intestinal cell renewal *in vivo* [154]. These data depict the possibility of the anti-aging effect of DCA or LCA in the mammalian or human intestine. In the liver, interestingly, neither DCA [155] nor LCA [156, 157] delayed aging, and did not promote aging, indicating that organ specificity is essential to the anti-aging effect of DCA and LCA. Ursodeoxycholic acid (UDCA) and tauroursodeoxycholic acid (TUDCA), used as a remedy for cholestasis, has attracted attention for its cytoprotective effect against ER stress [158], especially in the nervous system [138]. UDCA and TUDCA alleviate age-related changes and diseases such as Alzheimer’s disease [159], osteoarthritis [160], and cancer [161]. Notwithstanding the increase in progeroid mice [5], UDCA and TUDCA can also be regarded as anti-aging molecules worthy of further work.

Because of the risk of toxicity that certain BAs have, multiple trace BAs have captured the attention of scientists, proposed to be more effective and harmless. For example, 12-keto-chenodeoxycholic acid decreased in progeroid mice and recovered after fecal microbiota transplantation to prolong life [5]. However, vast amounts of BAs can be used to estimate anti-aging effects.

### 2.5 Short-Chain Fatty Acids

As key metabolites in the intestinal lumina, short-chain fatty acids (SCFAs) are fermented from resistant starch, dietary fiber, and other complex carbohydrates by a system of multiple microbes. The major SCFAs in the body, acetate, propionate, and butyrate, are mainly involved in physiological functions as follows: 1) energy metabolism, such as butyrate and propionate consumed in the intestine and liver, respectively; 2) histone deacetylases (HDAC) inhibitors; and 3) G protein-coupled receptor (GPCR) agonists, such as GPR43, GPR41, and GPR109A [162, 163]. SCFAs are implicated in a variety of neuropsychiatric disorders [164, 169], metabolic [170] and cardiovascular diseases [171], cancer [172, 173], and bone loss [174]. The fecal contents of total SCFA, consistent with acetate, propionate, and butyrate, are higher in centenarians than in those aged 80-90 years at the same area [130]. However, age-related decreases in serum acetate have been observed in Parkinson’s disease patients [175]. No age-related changes were shown by SCFA analysis of the Balb/c mouse cecal contents [176]. In progeroid mice, butyrate declines markedly [5, 134]. Further analysis to clarify the age-related tendency of SCFAs to set forth their anti-aging effects is required. It encourages studies on the anti-aging effect of SCFAs in a high-fiber diet, an efficient way to promote SCFAs, suppressed the central and peripheral inflammation caused by LPS common in the elderly [176].

The butyrate paradox that colorectal cancer is inhibited by butyrate but normal intestinal cells survive [177] is crucial to understanding the function of SCFAs in the intestinal tract. Butyrate is an energy source for colonocytes [162]. Meanwhile, SCFAs play a role as maintainers of intestinal homeostasis via regulation of autophagy [178], cell proliferation, and inflammation. For example, administration of a high-fiber diet reduces age-related colonic inflammation in mice fed with a low-fiber diet [176], which resulted partly from upregulated anti-inflammation factors such as age-related IL-10 [179] [180], as well as downregulated pro-inflammatory factors such as indoleamine 2,3-dioxygenase-1 (IDO-1) expression [181]. On the contrary, SCFAs facilitate immunological defense against pathogens by inducing antimicrobial peptide (AMP) production [182] and repairing intestinal tissue damaged by parasitic infection [183]. In addition, the age-induced breakage of intestinal permeability in mice is aggravated by the intake of SCFAs [176], resembling what was confirmed in the stressed mice because in part SCFAs contributed to rescuing the function of tight junctions diminished by stress [169]. In addition, increasing goblet cells [183] and bolstering Claudin-1, a tight junction protein [184], are involved in the protective effects of butyrate on the intestinal epithelial barrier.

The confusion comes up with deepening research on SCFAs in various organisms, besides the butyrate paradox in cancer. In contrast to a higher levels of fecal SCFAs in women with metabolic syndrome [185], active SCFA-producing bacteria are linked to lower hemoglobin A1c levels [170]. Meanwhile, SCFAs promoted exercise damage in Parkinson’s disease mice [166], however, improved clinical features in another drug-induced model mice [165]. The study also indicated a promoting nervous inflammation after treatment with SCFAs [166], in marked contrast to what is discussed above. Overall, it is noteworthy that the anti-aging effect of SCFAs varies in different species and animal models of a given species. Isobutyric acid, valeric acid, isovaleric acid, elevating in centenarians [130], also requires further study.

### 2.6 Tryptophan and Indoles

Tryptophan (Trp) is an essential aromatic amino acid. Dietary unabsorbed Trp follows three metabolic pathways in the intestine to kynurenine (Kyn), 5-HT, and indole derivatives [186]. Trp is prone to exert protection against aging as a kind of NAD precursor [187] although no evidence demonstrates the influence of it, to date. In the Kyn pathway, the key enzyme is IDO-1, which is thought to destroy the intestinal barrier by pro-inflammation [188, 181]. 5-HT basically acts on the ENS [186].

Produced by different bacterial strains with respective tryptophan enzymes, various indole derivatives participate in wide-ranged biological activities partly mediated by aryl hydrocarbon receptor (AhR) and PXR [186], further benefiting atherosclerosis [189], hypertension [190], fatty liver [191-194], tumor [195], and other age-related dysfunction. Indole extends the health span of *C. elegans* and *Drosophila* [196]. Moreover, dietary indole-3-carboxaldehyde (IAld) increased the survival rate of mice after total-body irradiation [196], validating the conservative protection effects of indoles. In the intestinal organoid system, IAld improves the proliferation of ISCs after damage through AhR [197]. Transcriptome analysis revealed that indole contributes to an increased expression of tight junctions in HCT-8 cells [198]. Emerging data showed indole acrylic acid (IA) and indole propionic acid (IPA) protect the intestinal epithelial barrier by enhancement of goblet cell function or moderation of inflammatory responses partly via PXR [50, 199-201]. Interestingly, both IA and IPA lie downstream of indole lactic acid (ILA), which *Bifidobacterium* species, a long-recognized probiotic genera [202], metabolizes tryptophan *in vitro* to produce only [203], denoting more benefits of those indoles that remain to be investigated.

### 2.7 Nicotinamide Adenine Dinucleotide and its Precursors

NAD is a vital coenzyme in all cells. As part of electron transfer, NAD participates in a vast body of internal reactions, particularly energy metabolism and sensing [187]. Three pathways guide five current, known precursors, Trp, nicotinic acid (NA), nicotinamide (NAM), nicotinamide riboside (NR), and nicotinamide mononucleotide (NMN) to NAD in cells [187]. Its nature as a substrate of poly-ADP-ribose-polymerases (PARPs) and sirtuins endows NAD with a target for aging anomalies [204]. In turn, aging witnesses a gradual depletion of cellular NAD in multiple tissues [205]. In goblet cells, *in vitro*, NAD treatment increases MUC2 expression, a major component of mucus [206]. A recent study showed that the provision of NR in drinking water reverses the age-related changes in the mouse intestine, such as the number of ISCs, formation of *in vitro* intestinal organoids, and recuperation from drug damage. The recovery of exacerbated ISCs is abrogated by the inhibitor of mTORC1 or SIRT1 [207]. This study sheds light on the benefits of the NAD/SIRT1/mTORC1 axis in the rejuvenation of the aging intestine. NAD and its replacement therapies deserve further investigation by well-designed clinical trials to validate the anti-aging value. Owing to the fact that not all of these precursors share consistent efficiency for conversion[208], the best oral NAD supplement strategy waits for our test. Besides, both inhibition of NAD consumption enzymes, such as PARP and CD38, and reinforcement of the key enzyme of NAD salvage pathways, NAM phosphoribosyltransferase (NAMPT), are theoretical options for improving NAD. Anti-CD38 antibodies are approved for use in multiple myeloma[209]. Moreover, intraperitoneal injection starting at the age of 26 months of NAMPT-containing extracellular vesicles purified from young mice is reported to restore movement activity and extend lifespan in elderly mice [210].

### 2.8 Urolithin A

Among the five products from the gut microbial fermentative activity of ellagitannin abundant in pomegranate as well as in nuts and berries, urolithin A (UA) is the hottest spot for its benefits in cancer and inflammation [211]. An increasing body mass index is followed by a decline in the distribution of UA, while a rising distribution of urolithin B. Similar changes occur to aging [212]. Treated with UA, *C. elegans* has a prolonged lifespan, and prevention or amelioration of age-related fitness decline, accompanied by activated mitophagy. However, in *mev-1* (associated with mitochondrial function) mutants, improvement of UA vanishes entirely. Furthermore, in rats and mice fed with UA daily, muscle function is promoted by the induction of mitophagy in the elderly. The power UA has to refit muscle and brain aging [213, 214] is shown to be involved in diverse biological processes such as antioxidation [215], autophagy [216], and ER stress [217]. In human skin fibroblasts, an Nrf2-dependent manner mediated UA’s antioxidative response to mitigate replicative senescence [218]. Through AhR/Nrf2, postinjury intake of UA protects mouse intestines from acute or chronic drug-induced damage by upregulation of tight junction proteins [219]. Moreover, the decrease in serum inflammatory markers [219] reveals that the gut protective effect of UA is attributed to suppression of systemic inflammation. Thanks to safety assessment guaranteeing the security of UA in clinical application [220], a promising avenue for UA intervention in the aging intestine comes into being.

**Table 2:** Mechanisms of strategies underlying anti-aging effects in intestine.

Strategy	Target	Mechanism	Ref
Diet restriction	Intestinal barrier	Enhancing gut barrier by upregulating MYC and rebalancing apoptosis	[99]
	ISCs	Enhancing stem cell competition to reduce mutation retention	[100]
Resveratrol	Immune system	Resisting inflammation by inhibiting NF-κB activation	[102]
	Intestinal barrier	Increasing tight junction proteins expression through PI3K/AKT pathway	[111] [112] [113]
	Morphological changes	-	[114]
Metformin	Intestinal microbiota	Altering microbial metabolism	[115] [118]
	Immune system	Resist colonic pathological inflammation by activating AMPK, inhibiting p53 activation	[119] [116]
	ISCs	Improving DNA damage and genomic instability by AKT/TOR signaling	[120] [121]
		Retarding ISCs aging by Atg6-depend autophagy	[122]
	Intestinal barrier	Increasing tight junction proteins expression through AMPK/JNK-dependent signaling	[123] [124] [125]
		Improving mucus by suppressing Wnt signaling to raise the number of goblet cells	[118]
NAD	Intestinal barrier	Improving mucus by increasing MUC2 expression	[206]
	ISCs	Improving ISC function by NAD/SIRT1/mTORC1 axis	[207]
Urolithin A	Intestinal barrier	Increasing tight junction proteins expression through AhR/Nrf2 pathway	[219]
Spermidine	Intestinal barrier	Reducing epithelial cell permeability by preserving location of tight junction proteins	[230]
		Increasing tight junction proteins in terms of synthesis and stability	[231]

ISC, intestinal stem cell; NF-κB, nuclear factor kappa B; PI3K, phosphatidylinositol-4,5-bisphosphate 3-kinase; AKT, protein kinase B; AMPK, adenosine-5’-monophosphate-activated protein kinase; TOR, target of rapamycin; Atg6, autophagy related 6; JNK, c-Jun N-terminal kinase; MUC2, Mucin 2; NAD, Nicotinamide adenine dinucleotide; SIRT1, Sirtuin 1; mTORC1, mammalian target of rapamycin complex 1; AhR, aryl hydrocarbon receptor; Nrf2, nuclear factor E2-related factor 2.

### 2.9 Spermidine

Spermidine (SPD), a natural polyamine, elicits its essential effects on cell growth, proliferation, and tissue regeneration [221]. SPD pool in mammals is contributed by dietary supply and synthesis of the intestinal microbiota [221], which suffer from an aging-related decline [222, 223] emphasizing the association between SPD and aging. Highly conserved ability of SPD is wildly reported to extend lifespan of *Saccharomyces*, *C. elegans*, *Drosophila* [224], mice [225], and human cells [224]. Restoration of autophagy, improvement of mitochondrial function, and reduction of ER stress are believed to be key for SPD to improve aging impairments, especially neurodegeneration [226], metabolic diseases [227], and cardiovascular and muscle-related disorders [225, 228, 229]. As a stimulus of T cell protein-tyrosine phosphatase, SPD rescues intestinal epithelial barrier dysfunction disrupted by inflammatory cytokine treatment in vitro [230]. It has been demonstrated in vitro that polyamine deprivation disturbs the synthesis and stability of a tight junction protein, occluding [231].

### Discussion

Unlike obvious wrinkles in the aged skin, senescence-associated deterioration in the intestine is too inconspicuous to draw people’s attention. However, the increased incidence of intestinal disease and morphological and functional changes of significance remind us of the damage of aging on the intestine. Notaly, progressive strategies help scientists trace slight modifications, especially dysbiosis, immune imbalance, stem cell exhaustion, barrier dysfunction, and enteric neurodegeneration, and further smooth out injury in the aged intestine.

Through the cooperation of sample analysis data and experimental results, several strategies are emerging for their anti-aging effects. In this review, we have appraised several strategies that we consider as candidates to postpone or avert the aged intestine. Ranging from diet control to pharmaceuticals and compounds metabolized by both host and microbiota, we reviewed DR and 8 kinds of compounds, which are listed in Table 2, except for BAs, SCFAs, and Trp for their large amounts of different derivatives to enumerate.

Among those means listed, we suggest that metformin should receive more focus as a highlighted strategy to rejuvenate the intestine under consideration of its power, covering comprehensive changes with the aged intestine. Metformin is a widely used antidiabetic drug for decades. After 2000, its pleiotropic effects beyond antidiabetic[232] have come to light. As mentioned above, we discuss the protective effect of metformin on intestine against aging. However, such anti-aging effects are mediated by multiple targets. The identification of a certain and integral action mode of metformin needs more studies. And further clinical data are also required to support metformin to be a treatment to rejuvenate the aged intestine in humans.

The search for potential strategies, especially special metabolites, to rejuvenate the aged intestine is ongoing. The fact that SCFAs amplify the function of AhR, a receptor of indoles[233], illuminates the cooperative effect of multiple dietary supplements. Hang et al.[234] selected 3-oxo-LCA and isoallo-LCA from nearly 30 types of BAs in the study, to search for the effect of BAs on the differentiation of immune cells[234], which inspired us to eliminate an integral screening process. However, it is worthwhile to consider several concepts. First, the age-related decreasing trend does not equal the anti-aging benefits. Moreover, aging in the intestine has many aspects. Confirmation *in vitro* does not always reappear *in vivo*. Concerning future directions, we highlight the convergence of deep metabolomic studies and experiments with speed and efficiency. It is anticipated to achieve a dozen well-received strategies to maintain the young intestine.
